# A machine and human reader study on AI diagnosis model safety under attacks of adversarial images

**DOI:** 10.1038/s41467-021-27577-x

**Published:** 2021-12-14

**Authors:** Qianwei Zhou, Margarita Zuley, Yuan Guo, Lu Yang, Bronwyn Nair, Adrienne Vargo, Suzanne Ghannam, Dooman Arefan, Shandong Wu

**Affiliations:** 1 Department of Radiology, University of Pittsburgh, Pittsburgh, PA 15213 USA; 2 College of Computer Science and Technology, Zhejiang University of Technology, Hangzhou, 310023 China; 3 Key Laboratory of Visual Media Intelligent Processing Technology of Zhejiang Province, Hangzhou, 310023 China; 4 Magee-Womens Hospital, University of Pittsburgh Medical Center, Pittsburgh, PA 15213 USA; 5 Department of Radiology, Guangzhou First People’s Hospital, School of Medicine, South China University of Technology, Guangzhou, 510180 China; 6 Chongqing Key Laboratory of Translational Research for Cancer Metastasis and Individualized Treatment, Chongqing University Cancer Hospital, Chongqing, 400030 China; 7 Department of Biomedical Informatics, University of Pittsburgh, Pittsburgh, PA 15213 USA; 8 Department of Bioengineering, University of Pittsburgh, Pittsburgh, PA 15213 USA; 9 Intelligent Systems Program, University of Pittsburgh, Pittsburgh, PA 15213 USA

**Keywords:** Translational research, Medical ethics, Radiography, Machine learning

## Abstract

While active efforts are advancing medical artificial intelligence (AI) model development and clinical translation, safety issues of the AI models emerge, but little research has been done. We perform a study to investigate the behaviors of an AI diagnosis model under adversarial images generated by Generative Adversarial Network (GAN) models and to evaluate the effects on human experts when visually identifying potential adversarial images. Our GAN model makes intentional modifications to the diagnosis-sensitive contents of mammogram images in deep learning-based computer-aided diagnosis (CAD) of breast cancer. In our experiments the adversarial samples fool the AI-CAD model to output a wrong diagnosis on 69.1% of the cases that are initially correctly classified by the AI-CAD model. Five breast imaging radiologists visually identify 29%-71% of the adversarial samples. Our study suggests an imperative need for continuing research on medical AI model’s safety issues and for developing potential defensive solutions against adversarial attacks.

## Abstract (web summary)

While active efforts are advancing medical AI model development and clinical translation, safety issues of medical AI models have emerged. Here, the authors investigate the effects on an AI model and on human experts of potential fake/adversarial images for breast cancer diagnosis.

## Introduction

Deep learning models have shown remarkable performance in many artificial intelligence (AI)-based medical applications, especially in medical image-related disease diagnosis and outcome prediction^1–4^. If deep learning algorithms continue to show a comparable or superior performance to human experts^5^, these AI tools will start to have a real role in clinical practice. Today, the community has recognized that it is imperative to build trustworthy, reliable, and safe AI systems for clinical deployment. One of the critical measures to the safety of an AI tool is to examine its behaviors under attacks from adversarial data^6–8^. The advancement of computational techniques, such as Generative Adversarial Networks (GANs), can generate adversarial data that may be intentionally used to attack AI models^9,10^. Under adversarial attacks, if a medical AI software makes a false diagnosis or prediction, it will lead to harmful consequences to patients, healthcare providers, and health insurances^11^. In the efforts of building trustworthy deep learning-based AI software for clinical applications, it is thus vital to investigate behaviors and protection of AI software under adversarial input data.

Adversarial attacks can be in different forms. Attacks made through adding sticker-like patches to images^12^ are often obvious to spot, because the added patches may break the image appearance consistency. Using images that are generated by GANs, or other methods^13^, is a more complicated form of attack, where imperceptible noises are usually added on images to induce a wrong output from an AI model. While the images with added adversarial noises may have undetectable visual appearance changes, this kind of attack may be easy for a domain expert to catch, because the induced wrong output does not match the visually unchanged diagnosis-sensitive contents of the images. In the current medical diagnosis context, radiologists routinely read or review images in daily practice, and this process can identify potential adversarial samples. Another example is attacks that generate entirely new/different images using standard GANs to replace original images. These fake images can also be identified by radiologists, who have access and use other sources of information (such as those in clinical notes) when viewing images, where data or clues, such as a patient’s personal history of diseases, previous diagnoses or anatomical assessment, historical or longitudinal imaging data, etc., can promptly alert radiologists that an entirely different image may be a fake. A more challenging scenario of adversarial attacks is to make intentional changes to specific anatomical or diagnosis-sensitive contents of the original images, aiming to simultaneously fool the diagnosis of an AI model and the visual inspection by human experts. Today, there are newly advanced GAN techniques that can generate highly plausible adversarial images by making targeted modifications to image contents. Examining the behaviors of an AI model under such highly plausible adversarial attacks represents a critical test for an AI software’s safety evaluation.

Deep learning has been shown to improve computer-aided diagnosis (CAD) of breast cancer on digital mammograms. Several recently reported deep learning-based AI-CAD models have shown promising performances^5,14–17^. Adversarial attacks to such AI-CAD models are emerging as a safety concern^11^ to patients, health providers, and legislation. Adversarial modifications to medical images can happen when the images are exposed to unauthorized access by hackers or malware^18^. Adversarial images, especially those modified with carefully designed manipulations, may generate unexpected, false, or wrong diagnostic results when fed to an AI-CAD model. In this work, we performed a study to evaluate an AI-CAD model’s behaviors under adversarial attacks of GAN-generated mammogram images by inserting cancerous tissue into normal images and by removing cancerous tissue in cancer-affected images. We also evaluated the effects of expert radiologists in visually identifying these kinds of adversarial images, without and with an educational intervention.

## Results

**Fig. 1:**
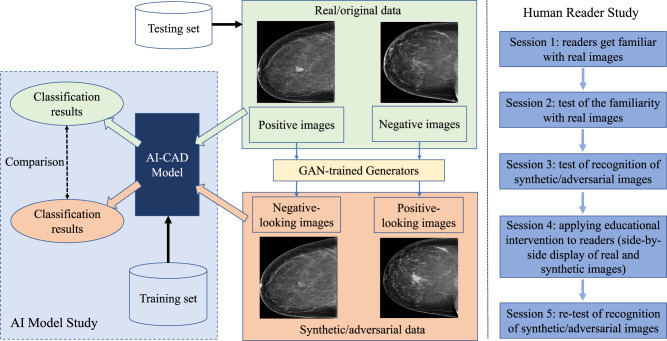
An overview of our study design. An AI-CAD model was first learned and then tested on the adversarial images generated by the GAN model which aimed to make modifications to the diagnosis-sensitive contents of images (by inserting or removing cancerous tissue). The reader study examined human experts’ capabilities to visually recognize the GAN-generated adversarial images.

Figure 1 illustrates an overview of our study design, which consists of two components: an AI model study and a human reader study. We assembled a study cohort composed of 1284 women from the University of Pittsburgh Medical Center and a total of 4346 mammogram images associated with this cohort. In this cohort, 918 patients were evaluated as negative (including benign findings) for breast cancer, while 366 patients were biopsy-proven positive for breast cancer malignancy. We first used a training set of the imaging data to build an AI-CAD model to distinguish cancer-positive cases vs. negative cases. We developed two GAN-based generators to synthesize adversarial samples from the real/original samples, where cancerous regions were “inserted” into negative images or “removed” from positive images by the GAN models, respectively. In the AI model study, we compared the effects of the AI-CAD model under the respective input of the original test set and the corresponding synthetic/adversarial test set to observe the model’s behaviors due to the adversarial attacks. In the human reader study, we recruited five breast imaging radiologists to visually observe images to identify potential adversarial samples (note, here, they are not required to make a positive vs. negative diagnosis on the images). We designed five sessions, including applying an educational intervention to assess the radiologists’ observation effects on adversarial images. The purpose of the AI model study was to investigate whether and to what extent the AI-CAD model may output a wrong diagnosis due to an adversarial input. The purpose of the human reader study was to measure whether and to what extent human radiologists may detect/capture an adversarial image through visual observation. Certified radiologists may be able to visually recognize fake/adversarial images using their medical knowledge and experience, especially if the GAN-generated images are less plausible (e.g., with obvious noises, introducing perceptible artifacts, incompliant to well-known anatomical structures, etc.). If radiologists can recognize that an image is fake, then they won’t make a diagnosis, or trust any diagnosis made on a fake image. In this sense, in a case where an adversarial input can fool an AI model and an automated detection of adversarial inputs is not in place, human experts’ visual observations may provide a realistic added protection by identifying potential adversarial inputs.

For considerations in computational efficiency and stability of the GAN models, we generated each adversarial image at two reduced resolutions: 1728 × 1408 (denoted as high resolution) and 1024 × 832 (denoted as low resolution). GAN models are known to be sensitive to image resolutions^19^ (it tends to collapse when dealing with high-resolution images). Note that in this study, 1728 × 1408 is considered a noticeable high resolution for GAN modeling, as in the literature most resolutions of GAN modeling ranged from 28 × 28 to 1024 × 1024^20–22^. We performed the AI model study and the human reader study with the images generated at both resolutions and compared the effects. Also note that for the human reader study, all five radiologists read all the images generated at the 1728 × 1408 resolution, while two radiologists (Readers 1 and 2) read the low-resolution (1024 × 832) images for comparison purposes (at this resolution we did not perform the readings with all five readers because of the limitation of the readers’ availabilities during the study period).

## AI-CAD model behavior under adversarial input

As shown in Fig. 1, we first built a deep learning-based AI-CAD model for classifying between breast cancer malignancy vs. normal/negative cases. The receptive field of this AI-CAD was optimally designed for mammogram images to cover the regions of most breast lesions in our dataset. The dataset was split at the patient level for the AI-CAD model training (80%), validation (10%), and testing (10%), respectively.

After training, we measured the model’s classification performance with respect to the original real test data and the corresponding GAN-generated adversarial counterparts from the real test data. We developed two GAN-trained U-Net^23^ models to generate the adversarial images. The mammogram images in the test set were modified with flipped labels by the GAN generators, which were trained to generate positive-looking fake counterparts from the negative mammogram images and to generate negative-looking fake counterparts from the positive mammogram images, respectively. Here, the fake counterpart images were intentionally assigned to a flipped label, that is, negative-looking fake counterparts were labeled as negative, and positive-looking fake counterparts were labeled as positive. The generated synthetic images from the real test set formed the adversarial test set, and the AI-CAD model’s classification was evaluated again using the adversarial test samples as input. We compared the model’s performance between the two test sets (namely, real images and adversarial images) to reveal whether, and to what extent, the AI-CAD model may be fooled by the fake/adversarial images. We used the area under the receiver operating characteristic curve (AUC) to measure the AI-CAD model’s classification performance. In general, if the model’s AUCs on both test sets are close, then it is more likely that the model has been largely fooled by the adversarial test set. We also further calculated classification accuracy to analyze the model’s behaviors with respect to subgroup testing samples of positive and negative cases.

Table 1 lists and compares the classification effects of the AI-CAD model on the test data at the two different resolutions. We first describe the results on the high-resolution (1728 × 1408) images. As can be seen, on the test set (74 real positive samples and 364 real negative samples), the AI-CAD model achieved an AUC of 0.82. The AUC was 0.94 when tested on the corresponding GAN-generated adversarial images (with flipped labels) of the test set. These two AUC values indicate that the AI-CAD model was largely fooled by the adversarial set of images. Furthermore, when using a threshold of 0.5 to calculate the classification accuracy, for the 74 real positive images, 44 (59.5%) were correctly classified as positive cases; and for their corresponding GAN-generated negative-looking fake counterparts (note that their labels were negative because of the flipping), 42 out of the 44 cases were classified as negative cases, which means that 95.5% (42 out of 44 cases) of the GAN-generated adversarial samples successfully fooled the classifier to wrongly output a negative diagnosis for these originally positive cases. Likewise, out of the 364 real negative images, 319 (87.6%) were correctly classified as negative cases; and for their corresponding GAN-generated positive-looking fake counterparts (note that their labels were positive because of the flipping), 209 out of the 319 cases were classified as positive cases, which means that 65.5% (209 out of 319 cases) of the GAN-generated adversarial samples successfully fooled the classifier to wrongly output a positive diagnosis for these originally negative cases. Putting the 44 positive and 319 negative cases together, 69.1% (i.e., 251 out of the 363 cases) of the GAN-generated adversarial images fooled the AI-CAD model.

**Table 1:** Classification effects of the AI-CAD model on the test data (74 real positive samples and 364 real negative samples, and their corresponding GAN-generated fake images) at two different resolutions.

Image resolution	AUC on real images (74 positive and 364 negative samples)	AUC on the corresponding GAN-generated fake counterparts (label flipped)	Classes of the real images	Percentage of correctly classified real images	Percentage of the fake counterparts (of the correctly classified real images) that fooled the AI-CAD model
1728 × 1408	0.82	0.94	Positive	59.5% (44/74)	95.5% (42/44)
			Negative	87.6% (319/364)	65.5% (209/319)
1024 × 832	0.82	0.79	Positive	58.1% (43/74)	88.4% (38/43)
			Negative	83.2% (303/364)	66.7% (202/303)

When looking at the results in Table 1 for the low-resolution (1024 × 832) images, we see a similar pattern as we did with the high-resolution images, where the GAN-generated adversarial samples fooled the AI-CAD classifier on 88.4% and on 66.7% of the cases for the positive and negative classes, respectively. Note that the AUC of the adversarial images was higher (0.94 vs. 0.79) for the high-resolution images, indicating that the high-resolution adversarial samples are more likely to fool the AI-CAD model to result in a wrong diagnosis.

## Human reader study to identify adversarial images

We designed a human reader experiment to evaluate the effects of recognizing/identifying potential GAN-generated adversarial images through visual observation by domain experts. For this experiment, we recruited five readers, including four attending radiologists (Readers 1 to 4) and one radiology fellow (Reader 5), all specializing in breast imaging. Readers 1–5 have 14, 13, 12, 7, and <1 year(s) of experience in breast imaging, respectively. All readers had no specific computational background or training on machine learning and GAN technique details. This experiment consisted of five sessions that were pre-designed before the experiment, and the key experimental design settings were blinded to the readers. The five readers were only informed that they were going to read some given digital mammogram images and were asked to give an assessment for each given image, using a 3-label scoring criterion:

1. this is a real mammogram image;
2. this is not a real mammogram image; or
3. unsure this image is real or not.

The readers were told to use a reasonable amount of time at their clinical discretion for assessing an image. For each session, the number of images were pre-assigned, the images were chosen randomly, and the exact same set of chosen images were distributed to corresponding folders for each reader to read. The readers were asked to complete the five sessions independently, without major/intentional interruptions. Also, in order to reduce potential complications, during the reading, they were not allowed to go back to read previous images (and change the assessment) once they moved on to read the next images.

The five sessions are described in the following.

**Fig. 2:**
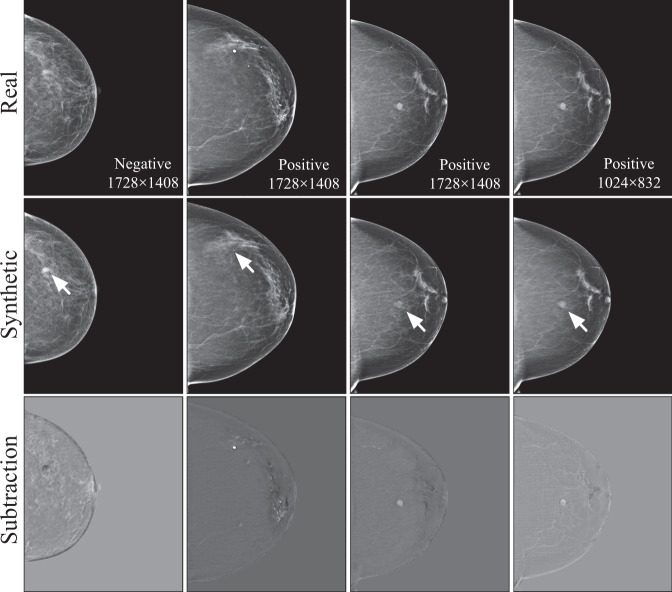
Examples of the images shown to the readers in the educational intervention. Each case consisted of a real image, the synthetic adversarial sample generated by the GAN model, and the difference calculated by the subtraction (real - synthetic) between the two images. Note that the arrows appeared in the second row were not part of the images shown for the educational purpose; they were provided here to indicate important changes made by the GAN models to the images.
- **Session 5**. After the educational intervention, Session 3 was repeated, but here all readers were given a completely different set of 440 images to read, where the 440 images consisted of half real images and half fake images. This session was designed to examine whether and how the readers’ performances in identifying fake images may have changed due to the educational intervention.

- **Session 1**. All readers were asked to observe 100 real mammogram images (readers knew the images were real). The purpose of this session was to have the reader become familiar with the real images in the setting of this study with the reduced resolutions.
- **Session 2**. All readers were given another set of 100 real mammogram images to read, but they were not told whether the images were real, fake, or a mixture. The purpose of this session was to assess, after Session 1, how well the readers were able to understand/read the real images in our study.
- **Session 3**. All readers were given 436 images to read, consisting of half real images and half fake/adversarial images. The readers were completely blinded to the labels (real or fake) of all the given images. We designed this session to examine how well the readers were able to identify the fake images from real images.
- **Session 4**. In this session, we applied an educational intervention to the readers. All readers were given 100 educational samples (Fig. 2) to read; here each educational sample consisted of a real image and its corresponding GAN-generated fake counterpart image, as well as the difference image (i.e., the subtraction of the real and synthetic image), displayed side-by-side. The purpose of this session was to educate the radiologists to potentially sense/learn the differences between the real and the GAN-generated fake images (or to perceive how the GAN generators had modified a real image to generate a fake/adversarial image). It should be noted that what we implemented here was a simple qualitative educational process for the readers, where no further information/explanation/guidance was provided to the readers, except showing the readers the real, fake, and subtraction images to observe. Nobody else was present/available to provide any assistance or consulting during this session. By viewing these images by themselves, it is each reader’s own experience and capability to potentially sense, perceive, or conceptualize/generalize the differences between the real and fake images they observed.

Table 2 summarizes the number of images used in each session. Except Session 4, the real images and their fake counterparts were all randomly selected without any overlapping among the sessions. Sessions 1 and 2 used images from the training set. Sessions 3 and 5 used images from a mixture of the validation and test sets. The real images in Session 4 were all randomly selected from the whole training set.

**Table 2:** Number of images used in different sessions in the human reader study (each sample in Session 4 consisted of one real image, its synthetic counterpart, and the difference/subtraction image).

Session #	Real images		GAN-generated fake images		Total
	Positive samples	Negative samples	Positive-looking counterparts	Negative-looking counterparts	
1	51	49	–	–	100
2	49	51	–	–	100
3	36	179	183	38	436
4	50	50	50	50	100
5	39	184	180	37	440

We reported and compared recognition accuracy of the readers across sessions. We calculated *p*-values to compare statistical significances of the accuracy values of a reader across Sessions 2, 3, and 5. Specifically, we split the samples in each session according to the reading order into several non-overlapped sub-groups of equal size to estimate the standard deviations, and the average of the standard deviations estimated for the three sessions was used to calculate one-sided *p*-values^24^.

### Reader’s performance on recognizing real high-resolution images

As shown in Table 3 for the study using high-resolution images, the results in Session 2 showed that all readers can recognize at least 50% of the real images, where two readers (Readers 1 and 3) had a much higher accuracy (i.e., 0.89 and 0.92) than the other three readers. When comparing the results between Session 3 and Session 2, all readers showed an increased accuracy in identifying the real images (all *p*-values < 0.03, except for Reader 2 where the *p*-value was 0.21). As the samples in Session 3 contained half fake images and all the samples in Session 2 were real images, these results showed that the presence of the fake images helped the readers in recognizing the real images; this is particularly obvious for Reader 4 (accuracy 0.6 for Session 2 vs. 0.94 for Session 3). Even though readers were not allowed to read images back and forth, they may still have learned some essential visual features of the fake/adversarial images and that possibly contributed to the process of identifying the real images. When comparing the results between Session 3 and Session 5, it showed that after the educational process, Readers 1 and 2 exhibited a significantly (*p*-values < 0.01) decreased recognition accuracy to the real images in Session 5, while Readers 3-5 showed no significant (all *p*-values > 0.09) changes in performance. This observation indicates that after being exposed to the comparative viewing of the paired real and GAN-generated fake images (in Session 4), it actually led to some confusion to the readers (especially for Readers 1 and 2), and thus reduced their confidence/capability in identifying the real images, where Readers 3-5 were relatively robust to the confusion. Considering the fact that Readers 4 and 5 have less clinical experience, their reduced performances may have to do with this fact and thus they may be less sensitive to this specific means of educational process.

**Table 3:** Accuracies of correctly identifying real/fake images at each session of the human reader study. Real images are the original images while fake images are the GAN-generated adversarial images.

Reader ID (number of years of clinical experience)	Mixed real and fake images	Real images (positive & negative cases)	Fake images (positive & negative cases)	Real images (positive cases)	Real images (negative cases)	Fake images (positive-looking images)	Fake images (negative-looking images)	Not sure an image is real or fake
High-resolution Images (1728 × 1408)								
Session 2								
Reader 1 (14)	–	0.89	–	0.88	0.90	–	–	0.05
Reader 2 (13)	–	0.60	–	0.59	0.61	–	–	0.00
Reader 3 (12)	–	0.92	–	0.96	0.88	–	–	0.03
Reader 4 (7)	–	0.60	–	0.67	0.53	–	–	0.00
Reader 5 (<1)	–	0.53	–	0.55	0.51	–	–	0.02
Session 3								
Reader 1 (14)	0.61	0.94	0.29	0.94	0.94	0.3	0.24	0.02
Reader 2 (13)	0.58	0.65	0.51	0.64	0.65	0.53	0.39	0.00
Reader 3 (12)	0.67	1.00	0.35	1.00	0.99	0.35	0.34	0.03
Reader 4 (7)	0.82	0.94	0.71	0.97	0.93	0.71	0.68	0.00
Reader 5 (<1)	0.53	0.61	0.44	0.61	0.61	0.43	0.5	0.01
Session 5								
Reader 1 (14)	0.58	0.85	0.30	0.72	0.88	0.32	0.22	0.06
Reader 2 (13)	0.57	0.49	0.65	0.31	0.53	0.72	0.32	0.00
Reader 3 (12)	0.75	0.97	0.51	0.97	0.97	0.49	0.59	0.03
Reader 4 (7)	0.82	0.95	0.70	1.00	0.93	0.67	0.84	0.00
Reader 5 (<1)	0.46	0.65	0.27	0.69	0.65	0.28	0.22	0.02
Low-resolution Images (1024 × 832)								
Session 2								
Reader 1 (14)	–	0.86	–	0.80	0.92	–	–	0.03
Reader 2 (13)	–	0.71	–	0.71	0.69	–	–	0.00
Session 3								
Reader 1 (14)	0.77	0.93	0.61	0.89	0.94	0.66	0.34	0.02
Reader 2 (13)	0.61	0.92	0.31	0.86	0.93	0.34	0.11	0.00
Session 5								
Reader 1 (14)	0.76	0.88	0.64	0.90	0.88	0.69	0.38	0.00
Reader 2 (13)	0.59	0.71	0.47	0.59	0.73	0.52	0.27	0.06

More specifically, when we look at the break-down accuracy of the positive/negative cases of the real images for each session, the accuracies of the positive cases (cancers) alone and negative cases (normal) alone were close to the accuracy of the combined set of positive and negative cases. This indicates that the readers’ reading effects are not sensitive to the samples’ classes (positive or negative). In addition, when comparing the break-down accuracy across sessions, we found that the patterns/trends of the accuracy changes from Session 2 to Session 3 and to Session 5 were similar to the patterns/trends on the combined set of positive and negative cases.

### Reader’s performance on recognizing fake/adversarial high-resolution images

The observations on recognizing the fake images are also summarized in Table 3. As can be seen from Session 3, there was a large range of accuracies among the five readers for the fake images (positive and negative cases combined), indicating that the readers’ initial abilities varied when facing the adversarial images, where their accuracies seemed not to be associated with the length of their clinical experience. After the educational intervention (Session 4), Readers 2, 3, and 5 showed significant (all *p*-values < 0.01) changes in accuracy in Session 5, where Readers 2 and 3 increased, but Reader 5 decreased, their performances; for Readers 1 and 4, they remained similar accuracies in Session 5 compared to Session 3.

When looking at the positive cases alone of the fake images, Reader 2 significantly increased accuracy (0.72 in Session 5 vs. 0.53 in Session 3; *p*-value < 0.01) and so did Reader 3 (0.49 in Session 5 vs. 0.35 in Session 3; *p*-value < 0.01). In contrast, Reader 5 significantly decreased accuracy (0.28 in Session 5 vs. 0.43 in Session 3; *p*-value < 0.01). When looking at the negative cases alone of the fake images, Reader 3 significantly increased accuracy (0.59 in Session 5 vs. 0.34 in Session 3; *p*-value < 0.01) and so did Reader 4 (0.84 in Session 5 vs. 0.68 in Session 3; *p*-value < 0.01). In contrast, Reader 5 showed a significantly decreased accuracy (0.22 in Session 5 vs. 0.50 in Session 3; *p*-value < 0.01). It can be seen that for Reader 2, the significant accuracy gain in the positive cases was at the cost of accuracy loss (from 0.39 to 0.32; insignificant, *p*-value = 0.13) in the negative cases; and likewise, for Reader 4, the significant accuracy gain in the negative cases was at the cost of accuracy loss (from 0.71 to 0.67; insignificant, *p*-value = 0.13) in the positive cases.

By comparing the overall results between the real images and fake images, it seems that the specific means of our educational intervention led the least experienced reader (i.e., Reader 5) to be incapable of correctly learning characteristics about the fake/adversarial images, thus resulting in the decreased recognition of the fake images after the education; but at the same time, the education did (slightly) increase this reader’s accuracy (0.65 in Session 5 vs. 0.61 in Session 3; *p*-value = 0.09) in recognizing the real images. For the other four readers, the overall comparisons indicate that the educational process enabled them to perform better in recognizing the fake/adversarial images, at no or little cost of losing some accuracy in recognizing the real images.

### Effects on the low-resolution images compared to high-resolution images

In Table 3, we also present the corresponding performances of the readers for the low-resolution images. As can be seen, similar reading behaviors/patterns of the two readers on the low-resolution images were observed in both the real and fake images when compared to the high-resolution images. The accuracy values of the two readers were also closer to each other on the low-resolution images, indicating less discrepancy on reading such images. The overall reading behaviors of the two readers were also consistent between reading the low- and high-resolution images. In addition, the readers’ accuracies on the low-resolution images were overall higher than those in the high-resolution images, indicating that it is more challenging for the readers to read the high-resolution adversarial images, which are more plausible to, and harder to distinguish from, the real images.

### Image reading time

Table 4 summarizes the time lengths each reader spent at each session. As can be seen, when using high-resolution images, Readers 1–3 had prolonged reading times for Session 5 compared to Session 3, indicating that after the educational intervention, these readers needed to spend more time to perceive/read an image in assessing whether the image was real or fake. For Readers 4 and 5, they had shortened times for Session 5 compared to Session 3. As mentioned in previous interpretations of the results, the educational session had almost no effect on Reader 4, which may or may not have to do with the observed shorter time that this reader spent on Session 5. In addition, Readers 2 and 5 spent much more time than other readers on Session 3 and Session 5; however, their overall reading accuracies are lower than the other three readers. This observation indicates that spending more time reading an image may not directly lead to improved performance in recognizing the fake/adversarial images. This may also imply that less experienced readers (e.g., Reader 5) may not have strong experience/knowledge to perceive the characteristics of our GAN-generated adversarial images, even if they spent more time in reading the images.

**Table 4:** Time spent in each session (unit: minute) of the reader study.

Reader	Session 1	Session 2	Session 3	Session 4	Session 5
High-resolution Images					
Reader 1	10	11	61	22	80
Reader 2	32	25	100	30	130
Reader 3	14	16	57	19	65
Reader 4	9	17	51	31	46
Reader 5	38	34	136	32	128
Low-resolution Images					
Reader 1	5	20	52	15	80
Reader 2	8	20	60	10	120

In Table 4 we also show the time lengths spent on the study when using the low-resolution images. As we can see, the two readers (Readers 1 and 2) spent less time than they did on the high-resolution images. This indicates that the high-resolution images are harder to read, and thus more time was used.

## Discussion

In this work we performed an evaluation study on the safety of a deep learning-based AI-CAD model for breast cancer diagnosis using mammography. The evaluation was based on adversarial attacks where fake mammogram images were synthesized by GAN models to mimic positive-looking and negative-looking images. In recent years, GAN models have been intensively studied in medical imaging applications^25^. Our study focused on using GAN models to generate highly plausible and high-resolution adversarial images with intentional modifications to insert or remove cancerous tissues. These kinds of adversarial images can lead to harmful consequences to mammogram-based AI-CAD models, because the adversarial modifications were made with respect to specific and diagnosis-sensitive contents in the original images, and thus it is even harder to detect such adversarial inputs than the common adversarial images perturbed with imperceptible noises^26–29^. Our main findings are that (1) the GAN-generated highly plausible adversarial images largely fooled the AI-CAD model in our experimental settings, and (2) the human readers (i.e., specialized breast imaging radiologists) visually recognized a certain proportion (accuracy range 29–71%) of the adversarial samples, but at the cost of sacrificing accuracy in recognizing real images. This indicates that the human readers failed to detect a substantial portion of the adversarial samples generated by our GAN models.

In the AI-CAD model study, it was important to build a model that had a reasonable performance (AUC at least > 0.80 based on our experience) for the diagnostic tasks–if a model has a very low classification performance (i.e., low AUC values) it will not be a good candidate to evaluate its effects under adversarial samples. Our AI-CAD model showed an AUC of 0.82, which means it was relatively accurate in classifying the positive/negative cases. In our high-resolution experiments, the GAN-generated fake images successfully fooled the AI-CAD model (i.e., generated a wrong diagnosis label) for 95.5% of the positive cases and for 65.5% of the negative cases in the testing set, meaning that the targeted modifications (i.e., removal or inserting of cancerous tissue) made by our GAN model led to wrong diagnostic results by the AI-CAD model. The advancements of modern GAN techniques have been showing surprising effects in generating fake/synthetic images. By inserting a lesion into an otherwise normal image to fool an AI model to give a wrong diagnosis of malignancy, or, similarly, by removing a lesion from an otherwise malignant image to fool an AI model to give a wrong diagnosis of normal, may lead to serious consequences for clinically deployed AI-CAD systems if these GAN models are used to generate adversarial images as input for AI-CAD models. It should be pointed out that such GAN models could possibly be trained using external/independent imaging data to perform adversarial attacks, as mammography is the standard, and widely available, imaging modality in the clinic.

For the human reader study, there are several important implications. While the educational intervention improved the recognition of the fake images for the more experienced readers, at a cost, it also reduced the recognition of the real images to some extent. This implies that the applied educational process can cause certain confusion to the readers in assessing the real images. For less experienced readers (e.g., Readers 4 and 5), the educational intervention seemed to be less helpful (in terms of the stable accuracy in recognizing the real images) or even harmful (in terms of the reduced accuracy after the educational intervention in recognizing the adversarial samples). This indicates that more clinical experience will be needed if we would have a human expert visually inspect the images before feeding them into an AI-CAD model. Or, this suggests that the simple educational intervention used in our study may not be optimal, especially for less experienced readers. Nevertheless, we suspect an effective educational intervention would still be a meaningful approach in educating radiologists to be aware of and to recognize adversarial samples. Through education, the improved recognition of fake images can reduce the chances of the AI-CAD model being fooled, which is more critical in real-world scenarios. The slightly reduced performance of recognizing real images may lead to some false alarms on real images, which may trigger additional workup to further examine these false alarm images, but the associated cost in time and labor efforts may be acceptably low. Hence, these kinds of false positives may be relatively tolerable in clinics, while benefiting from having a reduced number of false negatives in capturing adversarial attacks. We should point out, though, that in future work, it merits further studies on how to implement more effective educational interventions to educate radiologists to efficiently and accurately detect potential adversarial images.

In this study, we performed the experiments using adversarial data generated at two different resolutions. Overall, the AI-CAD model and the human readers showed similar behaviors/patterns on their performances at the two resolutions. Compared to the low-resolution images, the high-resolution images induced a higher chance of fooling the AI-CAD model and posed a higher challenge to the human readers in correctly identifying the adversarial images. It is well known that the computational stability of current GAN techniques is sensitive to image resolution. Along with the further development of new and advanced GAN models, it is possible that adversarial images generated with even higher resolutions may be more difficult to identify for both AI models and human readers.

Our study has some limitations that warrant future work. We only tested on one AI-CAD model, so our findings are specific to this model. However, the insights gained from this model will help us move forward to evaluate different technical implementations of the AI-CAD model, the GAN model, and other medical AI models. We used a small independent test set (10% of the full data) to examine the model’s behaviors, while we have a relatively large dataset. We did not use multi-fold cross-validation, as the goal was to examine the exact same model’s output for a given input with the original and the GAN-generated data. Further evaluations of our findings on large and external datasets will be important. In addition, other means of educational interventions are also worth evaluating in future work. And it will also be relevant to further assess how the adversarial samples may directly affect radiologists’ diagnostic performances.

While more efforts are advancing medical AI model development and clinical translation, more attention and research are now being placed on the safety aspects of AI models or systems. As discussed in a recent article^11^, motivations to commit adversarial attacks can include monetary gain, insurance fraud, temptation of favorable clinical trial outcomes, among others. Understanding the behaviors of an AI diagnostic model under adversarial attacks will help gain critical insights on identifying cybersecurity vulnerabilities and on developing mechanisms to potentially defend such attacks. At this stage, human experts remain a key role in detecting suspicious adversarial inputs, but effective educational interventions and tools are in great need. A potential scenario of adversary, AI, and radiologists may look like this: adversarial samples can fool AI models into making the wrong diagnosis (as shown in our study), and in that case, we would expect trained human radiologists to capture these fake/adversarial samples when visually reading/reviewing the images, where those identified suspicious images could then be further assessed to confirm and terminate the attacks. If, however, radiologists fail to fully identify such highly plausible fake/adversarial images, then this would constitute a real threat that must be addressed in clinical environments (e.g., developing defending solutions). Human-machine interactions may be one of the important approaches to address the safety issues of machine intelligence represented by the medical AI models, and further investigation in this area is much needed for the safety/security of future AI-augmented medicine. Our work is featured with both an AI model study and a human reader study, which represents a new contribution to the field, and we hope this will call for more research from the communities on this important topic.

In summary, we performed an evaluation study on an AI-CAD model and human readers pertaining to adversarial medical images. Our experiments showed that highly plausible adversarial samples can be generated on mammogram images by advanced GAN algorithms, and they can induce a deep learning AI model to output a wrong diagnosis of breast cancer. Certified human radiologists can identify such adversarial samples, but they may not be reliable to safely detect all potential adversarial samples, where an education process showed promise to improve their performance in recognizing the adversarial images. This poses an imperative need for continuing research on the medical AI model’s safety issues and for developing potential defensive solutions against adversarial attacks.

## Methods

This study received Institutional Review Board approval by the Human Research Protection Office (HRPO) at the University of Pittsburgh. Informed consent from patients was waived due to the retrospective nature. Verbal consent was obtained from the participants in the reader study. All five readers were women with ages of 41, 39, 44, 40, and 41 years old for Readers 1, 2, 3, 4, and 5 respectively. No compensation was provided to the readers for their participation in the reader study.

### Dataset

The patient cohort included 1284 women who underwent digital mammography screening for general populations from 2007–2014 at the University of Pittsburgh Medical Center. There were 918 patients who were evaluated as negative (including benign findings) of breast cancer and remained negative based on at least one year follow-up, and 366 patients who were biopsy-proven positive for breast cancer malignancy (consisting of 27% calcifications and 73% masses). Each patient had one full-field digital mammogram examination with up to four images, consisting of the left and right breast each with the craniocaudal (CC) and mediolateral oblique (MLO) view images. For the breast cancer positive cases, only images of the cancer-affected breast were used. For the negative cases, we used images of both breasts. There were a total of 4346 mammogram images included in this study. The original mammogram images have the bit-depth of 16, resolution of 4096 × 3328 or 3328 × 2560, and the pixel spacing of 0.07 mm. In our study we kept the original bit-depth but resized the original image resolutions to a consistent and ratio-preserved size of 1728 × 1408 (high-resolution images) and 1024 × 832 (low-resolution images) by the bi-cubic interpolation method. The reduction of image resolution was mainly for considerations on computational efficiency and stability of the GAN modeling processes.

### AI-CAD classifier

**Fig. 3:**
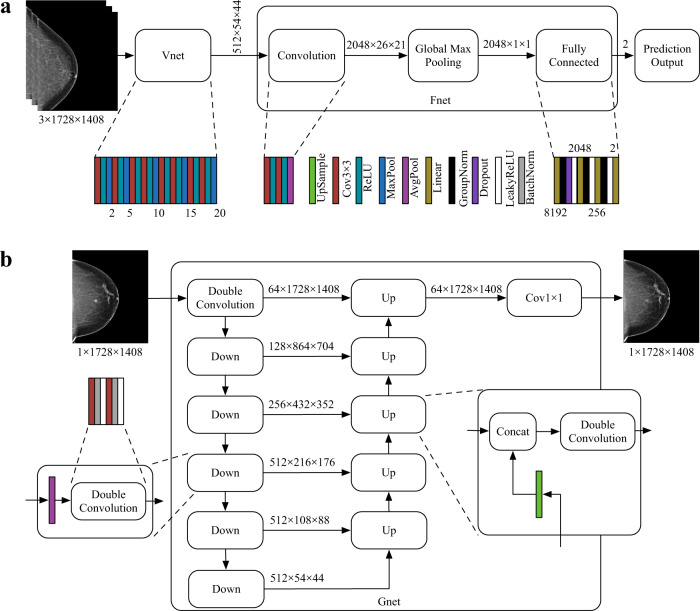
Network design of the models used in our study. The structure of the AI-CAD classifier (**a**) and the GAN generator (**b**).

Our AI-CAD model for classifying malignancy vs. normal/negative cases was implemented based on the VGG11 network^30^. As shown in Fig. 3a, before its first fully connected layer, the receptive field size was set to 406, which was calculated based on the statistics of our imaging dataset to cover most of the breast lesions. The optimal parameter value for the receptive field was to ensure that each element of the feature map before the last pooling layer can predict the presence of cancer-related features at the corresponding region in the input image. The first 20 layers of the VGG11 network (denoted by Vnet) were used to extract features. The rest of the AI-CAD model was denoted by Fnet, in which a Global Max Pooling layer was used to reduce the dimensionality. Because the size of lesions was relatively small, so only a limited number of the elements would be activated. That was why we used Global Max Pooling to magnify the output of the firing element rather than using Average Pooling to average the elements. Each GroupNorm layer had 32 feature groups. The drop rate of Dropout layers and the negative slope of LeakyReLU layers were both set to 0.2. The configuration of the AvgPool layer was: 2 × 2 kernel size, 1 stride, and 0 padding.

In training the AI-CAD model, each image was copied twice to fill the three channels of Vnet. We employed data augmentation to increase the size of the training samples, using transformations including vertical flipping, rotation (−45 to 45 degrees), scaling (factor = 0.5 to 2), and shearing (−45 to 45 degrees). Bilinear interpolation and zero value padding were used in these transformations. By using the same normalization method as VGG11, all images were normalized to the same range of intensity as a pre-processing for training.

The loss function we used in our model is shown in Eq. (1), where FL(·) denotes the focal loss^31^ and GP(·) denotes the gradient penalty^32,33^.

1 $${{{{{{\rm{Loss}}}}}}}_{{{{{{\rm{cls}}}}}}}={{{{{\rm{FL}}}}}}({p}_{{{{{{\rm{p}}}}}}})+{{{{{\rm{FL}}}}}}({p}_{{{{{{\rm{n}}}}}}})+{{{{{\rm{GP}}}}}}({g}_{{{{{{\rm{p}}}}}}},\,{g}_{{{{{{\rm{n}}}}}}});$$

2 $${{{{{\rm{FL}}}}}}({p}_{s})=-\frac{1}{N}\mathop{\sum }\limits_{j=1}^{N}{(1-{p}_{j})}^{\gamma }\log ({p}_{j});$$

3 $${{{{{\rm{GP}}}}}}({x}^{1},{x}^{2})=\frac{k}{2N}\left(\mathop{\sum }\limits_{j=1}^{N}\left({\left\Vert \nabla {{{{{\rm{Fnet}}}}}}\left({{{{{\rm{Vnet}}}}}}({x}_{j}^{1})\right)\right\Vert }_{2}^{o}+{\left\Vert \nabla {{{{{\rm{Fnet}}}}}}\left({{{{{\rm{Vnet}}}}}}({x}_{j}^{2})\right)\right\Vert }_{2}^{o}\right)\right).$$

here, *g*_p_ and *g*_n_ denotes positive and negative images in one batch, respectively; *p*_p_ or *p*_n_ represents the probability output of the SoftMax function for the positive classification of *g*_p_ or the negative classification of *g*_n_; *p*_*s*_ can be *p*_p_ or *p*_n_, in which there are *N* predicted probabilities (*N* is the batch size and *N* = 4); *p*_*j*_ denotes the predicted probability of the *j*-th image; *k* and *o* are the coefficient (*k* = 10) and power (*o* = 4) of the gradient penalty, which were empirically determined; $\gamma$ is the focusing parameter ($\gamma$ = 2) of the focal loss.

The Adam optimizer^34^ was used to train Vnet and Fnet, with the following parameters: exponential decay rates *β*_1_ = 0.5 and *β*_2_ = 0.9, learning rate 0.0001 for Fnet and 0.00001 for Vnet. Note that because Vnet was initialized by VGG11, we started the training by only optimizing Fnet in order to maintain the initialized weights in Vnet, and after a certain number of epochs (here it was 41 in our study), both Vnet and Fnet were trained simultaneously.

### GAN model generator and discriminator

The GAN model consists of a generator and a discriminator. The generator was trained to generate adversarial/fake images. The generator was implemented based on U-Net^23^, as shown in Fig. 3b (denoted by Gnet). The AvgPool layers were used with 2 × 2 kernel and stride 2 to down-sample feature maps, while the UpSample layers were used with scale factor 2 and bilinear interpolation to up-sample feature maps. Other layers used similar configurations as the networks used in the AI-CAD classifier. The input of Gnet was a single-channel normalized image by using the normalization method of the first channel of VGG11. The output of Gnet used Sigmoid (1/(1 +e^-*x*^)) to normalize its pixel value into the range from 0 to 1.

The discriminator of the GAN model had the same structure as the AI-CAD classifier (Fig. 3a), except the last fully connected layer of Fnet was modified to output the scores of real or fake images, using only one output neuron. The discriminator is denoted by Dis(·) = Dnet(Vnet(·)) where Dnet refers to the modified Fnet. To accelerate the training of the GAN model, the discriminator was initialized by the trained AI-CAD classifier, namely, Fnet(Vnet(·)). Note that the implementation of the discriminator also used VGG11. VGG11 was a pre-trained network without batch normalization, and the use of VGG11 here was aligned to a previous literature^35^ that suggested not to use batch normalization for GAN model training.

In training the GAN model, we used the same data augmentation and sampling strategy as the classifier. The training process was similar for generating fake images for the positive cases and negative cases, but it was performed independently. For the sake of clarity, here we only describe the training method for generating negative-looking fake images from real positive images. Eqs. (4)–(8) show the loss functions for training the GAN model. When training for generating positive-looking fake images, we simply switched the related variables in these equations.

4 $${{{{{{\rm{Loss}}}}}}}_{{{{{{\rm{dis}}}}}}}=-{{{{{\rm{Dis}}}}}}(x)+{{{{{\rm{Dis}}}}}}({y}_{{{{{{\rm{fn}}}}}}})+{|{{{{{\rm{Dis}}}}}}(x)-1|}_{1}+{{{{{\rm{GP}}}}}}(x);$$

5 $${{{{{\rm{Dis}}}}}}(g)=-\,{{{{\mathrm{ln}}}}}\left(1+{e}^{-{{{{{\rm{Dnet}}}}}}({{{{{\rm{Vnet}}}}}}(g))}\right);$$

6 $${{{{{\rm{GP}}}}}}(x)=\frac{k}{2}{\left\Vert \nabla {{{{{\rm{Dnet}}}}}}({{{{{\rm{Vnet}}}}}}(x))\right\Vert }_{2}^{o};$$

7 $${{{{{{\rm{Loss}}}}}}}_{{{{{{\rm{gen}}}}}}1}={\left\Vert {y}_{{{{{{\rm{fn}}}}}}}-y\right\Vert }_{2}^{2}/{\left\Vert y\right\Vert }_{2}^{2};$$

8 $${{{{{{\rm{Loss}}}}}}}_{{{{{{\rm{gen}}}}}}2}=-{{{{{\rm{Dis}}}}}}({y}_{{{{{{\rm{fn}}}}}}})+{{{{{{\rm{Loss}}}}}}}_{{{{{{\rm{gen}}}}}}1};$$

where *x* denotes the negative images and *y* denotes the positive images; *y*_fn_ denotes the 3-channel pseudo color images that were generated from the output of Gnet(*y*); *g* can be *x*, *y* or *y*_fn_; Loss_dis_ is the discriminator loss for training the discriminator; GP(*x*) is the gradient penalty in which the coefficient *k* and the power *o* were experimentally set to 10 and 4, respectively; Loss_gen1_ is the identical loss that trains Gnet to reconstruct its input; Loss_gen2_ is the adversarial loss that trains Gnet to fool the discriminator.

Based on the common choice of the loss function for GAN model training^34^, we added a stabilizer |Dis(*x*)-1|_1_ in the loss function of our discriminator (Eq. (4)) to prevent the discriminator training from divergence. Considering that the lesion size is small relative to the size of the breast, we added the identical loss Loss_gen1_ to the adversarial loss of the generator (Eq. (8)) to improve the fidelity of the generator.

**Fig. 4:**
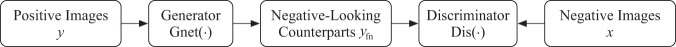
The GAN model training procedures for generating negative-looking fake/adversarial images. The procedures (not depicted here) were similar for generating positive-looking fake/adversarial images.

The configuration of the optimizer for training the GAN model was Adam^34^ with exponential decay rates *β*_1_ = 0.5 and *β*_2_ = 0.9. Gnet and the last layer of Dnet were initialized with random weights, and they were trained separately in order to not interfere each other. First, Gnet was trained with learning rate 0.0001 and Loss_gen1_ (Eq. (7)) until Loss_gen1_ on the training dataset decreased to be less than 0.002. Second, the last layer of Dnet was trained with learning rate 0.0001 and Loss_dis_ (Eq. (4)) where Gnet and the rest parts of Dnet were involved but not trained; the training stopped when Dis(*x*)-Dis(*y*_fn_) increased to be greater than 0.2. In order to reduce potential overfitting, the training for Gnet and Dnet was both iterated for a small number of epochs (i.e., 11 for Gnet and 10 for Dnet). After that, all the networks (as depicted in Fig. 4) were trained using the following typical configurations: training Gnet once every 5 batches with Loss_gen2_, and learning rate 0.00001, to minimize the difference between a negative-looking counterpart and a negative image; training Dnet with Loss_dis_, learning rate 0.0001 for Fnet, and 0.00001 for Vnet, to score negative images to be higher than negative-looking counterparts. The GAN training stopped when Dis(*x*)-Dis(*y*_fn_) reached to less than 0.01.

The software that we used for implementing our methods and data analysis included Python (3.7.4), Pytorch (1.7.1), CUDA (11.1), NumPy (1.16.2), Pillow (5.4.1), and Commercial MATLAB R2020b (9.9.0.1467703) 64-bit (win64).

### Reporting summary

Further information on research design is available in the Nature Research Reporting Summary linked to this article.

## Supplementary information

Reporting Summary

## Data Availability

The imaging data used in this study are not publicly available because they may contain private patient health information. Interested users may request access to these data for research purposes, through contacting the corresponding author. Institutional approvals of data sharing will be required along with signed data use agreements and/or material transfer agreements, where the data use conditions/restrictions will be negotiated based on the purposes of the data requests. Derived results reported in this paper and supporting the findings of this study are available upon requests.

## Code availability

The source code of the developed models and algorithms in this study has been deposited at GitHub: https://github.com/QianWeiZhou/Medical-AI-Adversarial-Attack.
