# Supplementary material for: A machine and human reader study on AI diagnosis model safety under attacks of adversarial images
Source: Nat Commun. 2021 Dec 14;12:7281. doi: 10.1038/s41467-021-27577-x (PMC8671500; doi:10.1038/s41467-021-27577-x)
Supplement: Supplementary file 1 — Reporting Summary [file 41467_2021_27577_MOESM1_ESM.pdf]

nature portfolio

Corresponding author(s): Shandong Wu

Last updated by author(s): Nov 19, 2021

Reporting Summary

Nature Portfolio wishes to improve the reproducibility of the work that we publish. This form provides structure for consistency and transparency in reporting. For further information on Nature Portfolio policies, see our Editorial Policies and the Editorial Policy Checklist.

Statistics

For all statistical analyses, confirm that the following items are present in the figure legend, table legend, main text, or Methods section.

n/a	Confirmed	
☐	☒	The exact sample size (*n*) for each experimental group/condition, given as a discrete number and unit of measurement
☒	☐	A statement on whether measurements were taken from distinct samples or whether the same sample was measured repeatedly
☐	☒	The statistical test(s) used AND whether they are one- or two-sided<br>*Only common tests should be described solely by name; describe more complex techniques in the Methods section.*
☐	☒	A description of all covariates tested
☒	☐	A description of any assumptions or corrections, such as tests of normality and adjustment for multiple comparisons
☐	☒	A full description of the statistical parameters including central tendency (e.g. means) or other basic estimates (e.g. regression coefficient) AND variation (e.g. standard deviation) or associated estimates of uncertainty (e.g. confidence intervals)
☐	☒	For null hypothesis testing, the test statistic (e.g. *F*, *t*, *r*) with confidence intervals, effect sizes, degrees of freedom and *P* value noted<br>*Give P values as exact values whenever suitable.*
☒	☐	For Bayesian analysis, information on the choice of priors and Markov chain Monte Carlo settings
☒	☐	For hierarchical and complex designs, identification of the appropriate level for tests and full reporting of outcomes
☒	☐	Estimates of effect sizes (e.g. Cohen's *d*, Pearson's *r*), indicating how they were calculated

Our web collection on statistics for biologists contains articles on many of the points above.

Software and code

Policy information about availability of computer code

Data collection	An internally validated in-house program was used for de-identification of imaging data. Commercial MATLAB R2020b (9.9.0.1467703) 64-bit (win64) was used for image pre-processing.
Data analysis	We used Python (3.7.4), Pytorch (1.7.1), CUDA (11.1), NumPy (1.16.2), Pillow (5.4.1), and Commercial MATLAB R2020b (9.9.0.1467703) 64-bit (win64) to analyze the data. For custom code or algorithm, please refer to the Method section. The source code of the developed models and algorithms in this study has been deposited at GitHub: https://github.com/QianWeiZhou/Medical-AI-Adversarial-Attack.

For manuscripts utilizing custom algorithms or software that are central to the research but not yet described in published literature, software must be made available to editors and reviewers. We strongly encourage code deposition in a community repository (e.g. GitHub). See the Nature Portfolio guidelines for submitting code & software for further information.

Data

Policy information about availability of data

All manuscripts must include a data availability statement. This statement should provide the following information, where applicable:

- Accession codes, unique identifiers, or web links for publicly available datasets
- A description of any restrictions on data availability
- For clinical datasets or third party data, please ensure that the statement adheres to our policy

The imaging data used in this study are not publicly available because they may contain private patient health information. Interested users may request access to these data for research purposes, through contacting the corresponding author. Institutional approvals of data sharing will be required along with signed data use agreements and/or material transfer agreements, where the data use conditions/restrictions will be negotiated based on the purposes of the data requests. Derived results reported in this paper and supporting the findings of this study are available upon requests.

Field-specific reporting

Please select the one below that is the best fit for your research. If you are not sure, read the appropriate sections before making your selection.

- [x] Life sciences
- [ ] Behavioural & social sciences
- [ ] Ecological, evolutionary & environmental sciences

For a reference copy of the document with all sections, see nature.com/documents/nr-reporting-summary-flat.pdf

Life sciences study design

All studies must disclose on these points even when the disclosure is negative.

Sample size	No formal sample size calculation was performed due to the nature of this study. The dataset size and size of human readers were determined based on experience of the researchers and they were deemed appropriate for a proof-of-concept study. The imaging data of this study are from the cohorts that had been collected/curated in our previous studies on breast cancer.
Data exclusions	Imaging data were excluded if they have poor imaging quality that is not acceptable in clinical settings.
Replication	We performed our study (both the AI model evaluation and human reader study) on images with two different resolutions to examine the reproducibility of our study and to evaluate the potential influence of image resolutions to our study goals. These attempts at replication were successful.
Randomization	When we generated image samples for the five reading sessions, images are randomly selected from a data pool and assigned to the sessions.
Blinding	The investigators were blinded to group allocation during data collection. The human readers are blinded to the diagnosis model and GAN model development, study design, and data analysis of their reading results. This is to minimize potential influence to the readers for performing the reading sessions. The diagnosis groups (positive or negative cases) and the reading sessions were made blinded to all authors, except the first author who needed the group information in performing the analysis of the results.

Reporting for specific materials, systems and methods

We require information from authors about some types of materials, experimental systems and methods used in many studies. Here, indicate whether each material, system or method listed is relevant to your study. If you are not sure if a list item applies to your research, read the appropriate section before selecting a response.

Materials & experimental systems

n/a	Involved in the study
x	Antibodies
x	Eukaryotic cell lines
x	Palaeontology and archaeology
x	Animals and other organisms
	x Human research participants
x	Clinical data
x	Dual use research of concern

Methods

n/a	Involved in the study
x	ChIP-seq
x	Flow cytometry
x	MRI-based neuroimaging

Human research participants

Policy information about studies involving human research participants

Population characteristics	The imaging data of this study are from the cohorts that had been collected/curated in our previous studies on breast cancer. The patient cohort of this study included 1,284 women who underwent digital mammography screening for general populations from 2007-2014 at a single medical center. There were 918 patients who were evaluated as negative (including benign findings) of breast cancer and remained negative based on at least one year follow-up, and 366 patients who were biopsy-proven positive for breast cancer malignancy (consisting of 27% calcifications and 73% masses). The readers in the human reader study include 5 radiologists with 14, 13, 12, 7, and <1 year(s) of experience in breast imaging specialty, respectively. All the readers are women with ages of 41, 39, 44, 40, and 41 years old for Readers 1, 2, 3, 4, and 5 respectively. No compensation was provided to the readers for their participation in the reader study.
Recruitment	The readers in this study were recruited through directly approaching or sending solicitation emails to the clinical collaborators (i.e., breast imaging radiologists in our institution) or laboratory members. The selection of the participating readers may bring in self-selection bias because some readers were associated collaborators of the laboratory of the corresponding author. This may not capture the full capabilities of human readers in reading the adversarial images.
Ethics oversight	This study received Institutional Review Board approval by the Human Research Protection Office (HRPO) at the University of Pittsburgh. Informed consent from patients was waived due to the retrospective nature. Verbal consent was obtained from the participants in the reader study.

Note that full information on the approval of the study protocol must also be provided in the manuscript.
